# Molecular Cloning and Functional Characterization of Tibetan Porcine STING

**DOI:** 10.3390/ijms13010506

**Published:** 2012-01-04

**Authors:** Zhiqing Huang, Xiaoling Chen, Keying Zhang, Bing Yu, Xiangbing Mao, Ye Zhao, Daiwen Chen

**Affiliations:** Key Laboratory for Animal Disease-Resistance Nutrition of Sichuan Province, Institute of Animal Nutrition, Sichuan Agricultural University, Yaan, Sichuan 625014, China; E-Mails: zqhuang@sicau.edu.cn (Z.H.); xlchen@sicau.edu.cn (X.C.); zkeying@yahoo.com (K.Z.); bingyucn@yahoo.com (B.Y.); xiangbingm@hotmail.com (X.M.); ye_zhao@yahoo.com (Y.Z.)

**Keywords:** cloning, innate immunity, IPEC-J2 cells, Tibetan porcine STING, type I interferon

## Abstract

Tibetan pig is well known for its strong disease resistance. However, little is known about the molecular basis of its strong resistance to disease. Stimulator of interferon (IFN) genes (STING), also known as MPYS/MITA/ERIS/TMEM173, is an adaptor that functions downstream of RIG-I and MAVS and upstream of TBK1 and plays a critical role in type I IFN induction. Here we report the first cloning and characterization of STING gene from Tibetan pig. The entire open reading frame (ORF) of the Tibetan porcine *STING* is 1137 bp, with a higher degree of sequence similarity with Landrace pig (98%) and cattle (88%) than with chimpanzee (84%), human (83%) or mouse (77%). The predicted protein is composed of 378 amino acids and has 4 putative transmembrane domains. Real-time quantitative PCR analysis indicated that Tibetan pig *STING* mRNA was most abundant in the lung and heart. Overexpression of Tibetan porcine STING led to upregulation of *IFN-β* and IFN-stimulated gene 15 *(ISG15)* in porcine jejunal epithelial cell line IPEC-J2 cells. This is the first study investigating the biological role of STING in intestinal epithelial cells, which lays a foundation for the further study of STING in intestinal innate immunity.

## 1. Introduction

Innate immunity is the first line of host defense against invading pathogens. Innate immune responses are initiated by the host’s pattern recognition receptors (PRRs), which recognize conserved pathogen-associated molecular patterns (PAMPs) of microorganisms [1–3]. Upon recognition, PRRs trigger a series of signaling events leading to the expression of type I interferons (IFNs), IFN-stimulated genes (ISGs) and proinflammatory cytokines, which are all critical for the protection of a host suffering from microbial infection [4]. The host has developed at least two distinct mechanisms for the recognition of viral RNAs, which represents the first step of innate antiviral response [5]. One is mediated by Toll-like receptors (TLRs), such as TLR3, which recognizes viral double-stranded RNA (dsRNA) released by infected cells [6]. The other mechanism involves the RNA helicases RIG-I and MDA5, which function as intracellular viral RNA sensors [7,8].

Stimulator of IFN genes (STING), also known as TMEM173/MPYS/MITA/ERIS, has recently been shown to be an adaptor that functions downstream of RIG-I and MAVS and upstream of TBK1 [9,10]. Overexpression of STING activates interferon regulatory factor 3 (IRF3), leads to upregulation of type I IFNs as well as ISGs expression, and inhibits viral replication, whereas knockdown of endogenous STING has the opposite effects [11–13]. These results demonstrate that STING is a key component in the virus-triggered IRF3 activation pathway and cellular antiviral response and plays a critical role in type I IFN induction.

Tibetan pig is a special Chinese indigenous pig breed, which is distributed in the Qinghai-Tibet Plateau at altitudes ranging from 2200 to 4400 m. It is well known for its remarkably strong disease resistance compared to other pig breeds. In this study, we cloned the Tibetan porcine *STING* cDNA and examined its tissue distribution. We also examined the effects of STING on *IFN-β* and *ISG15* expressions at mRNA levels.

## 2. Results and Discussion

### 2.1. Cloning and Sequence Analysis of Tibetan Porcine STING

Based on the sequence of Landrace pig *STING* (GenBank ID: FJ455509), the primers pMD19-T-STING-F and pMD19-T-STING-R were designed and used to amplify the potential *STING* cDNA sequence from total RNA extracted from skeletal muscle of Tibetan pigs. The entire open reading frame (ORF) of Tibetan porcine *STING* contains 1137 bp (GenBank ID: JN226147) and encodes 378 amino acid residues (Figure S1). This nucleotide sequence shares 77%, 83%, 84%, and 88% homology with the known STING sequences of mouse (GenBank ID: NM_028261), human (GenBank ID: NM_198282), chimpanzee (GenBank ID: XM_001135484), and cattle (GenBank ID: NM_001046357), respectively. Not surprisingly, the nucleotide sequence of STING in the Tibetan pig is nearly identical to that of Landrace pig reported by Xie *et al*. [14], with 12 nucleotide differences. Whether some of these 12 differences may be due to single nucleotide polymorphisms need to be identified in future investigations.

**Figure 1:**
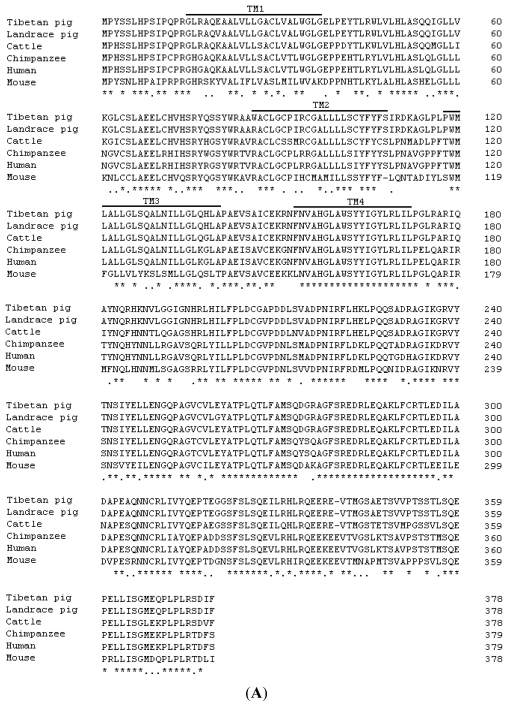

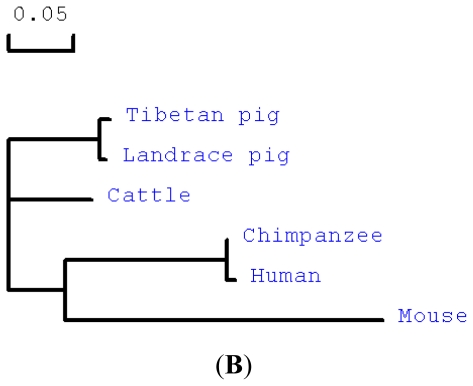
Alignment, phylogenetic tree, and architecture of the predicted amino acid sequence for Tibetan porcine STING. (**A**) Multiple comparison of amino acid sequences of Tibetan pig, Landrace pig, chimpanzee, cattle, human and mouse STING. Four putative transmembrane regions (TM) of Tibetan porcine STING are indicated by bold lines. The numbers on the right of every line refer to positions of the amino acid residues; (**B**) Phylogenetic analysis of Tibetan porcine STING. The neighbor-joining tree was constructed by DNAMAN. The sequences were derived from the predicted amino acid sequences of Tibetan porcine STING (AEL97644) and the GenBank entries with accession numbers ACJ70708 (Landrace pig), NP_001039822 (cattle), XP_001135484 (chimpanzee), NP_938023 (human), and NP_082537 (mouse). The scale bar is 0.05.

The predicted amino acid sequence of the Tibetan porcine STING was compared with mammalian STING amino acid sequences available from GenBank (Figure 1A). There was 69%, 76%, 77% and 86% similarity to that of mouse (NP_082537), human (NP_938023), chimpanzee (XP_001135484) and cattle STING (NP_001039822), respectively. Not surprisingly, the Tibetan porcine STING protein sequence (AEL97644) had 98.68% homology with known Landrace pig sequence (ACJ70708), with 5 amino acid substitutions at residues 20 (Glu-Val), 86 (Trp-Arg), 208 (Ala-Val), 224 (Lys-Glu) and 260 (Glu-Gly). Structural analysis with the SMART program (http://smart.embl-heidelberg.de/smart/set_mode.cgi?NORMAL=1) showed that Tibetan porcine STING contains four putative transmembrane (TM) domains (aa 15–37, aa 86–108, aa 118–140, and aa 153–172) at its N-terminus (Figure 1A). Similar transmembrane domains have also been reported in the STING sequences of human [13] and Landrace pig [14] and can be predicted in the mouse STING sequence, suggesting that transmembrane domains may be the common features of mammalian STING. In addition, the transmembrane domains of human STING have been demonstrated to be critical to retain this protein on the membrane of endoplasmic reticulum or mitochondria [12,13]. As mentioned above, there were 5 amino acid differences between Landrace and Tibetan porcine STINGs. Among them, an amino acid substitution at residues 20 (Glu-Val) was observed in its transmembrane domains. Whether this substitution may lead to the differences in disease resistance between Landrace and Tibetan pigs need further investigation. A phylogenetic analysis of the amino acid sequence was performed, and the resulting neighbor-joining tree showed that the Tibetan pig has a closer genetic relationship with Landrace pig and cattle than with chimpanzee, human and mouse (Figure 1B).

### 2.2. Tissue Distribution of the Tibetan Porcine STING mRNA

**Figure 2:**
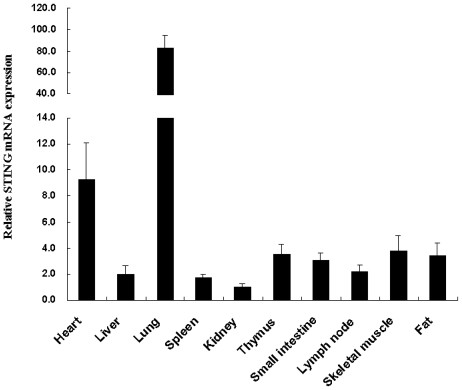
Relative mRNA expression of Tibetan porcine *STING* in different tissues. Total RNA from different tissues of 3 healthy Tibetan pigs was used to perform the real-time quantitative PCR. Samples were performed in duplicate. The amount of Tibetan porcine *STING* mRNA was normalized to the amount of *β-actin* mRNA. Data are presented as mean ± SE (*n* = 3), in arbitrary units.

The mRNA expression profile of Tibetan porcine *STING* was determined by real-time quantitative PCR in various tissues. As shown in Figure 2, Tibetan porcine *STING* transcripts were most abundant in the lung and heart, followed by the skeletal muscle, thymus, fat, small intestine, lymph node and liver, and to a lesser extent in the spleen and kindey. The tissue distribution pattern of STING has been previously examined in several mammals, including human [11,13], mouse [12] and Landrace pig [14]. Landrace pig *STING* mRNA was mainly expressed in the spleen, lymph node and lung [14], while Tibetan pig *STING* mRNA was mainly detected in the lung and heart in the present study. The reason for this discrepancy may be due to the different age and health status of the animals. In addition to the lung and heart, Tibetan porcine *STING* was also detected in all other examined tissues, indicating that Tibetan porcine STING may be important in the innate immune system in various organs of the pigs.

### 2.3. Overexpression of Tibetan Porcine STING Leads to Upregulation of IFN-β and ISG15 in IPEC-J2 Cells

**Figure 3:**
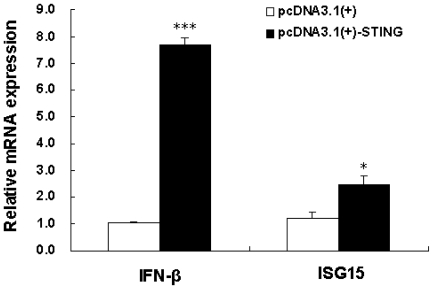
Overexpression of Tibetan porcine STING leads to upregulation of IFN-β and ISG15. IPEC-J2 cells were transfected with 2 μg/well of the plasmid pcDNA3.1(+)-STING or the empty vector pcDNA3.1(+). *IFN-β* and *ISG15* mRNA levels were determined using real-time quantitative PCR 24 h after transfection. The amount of *IFN-β* and *ISG15* mRNA was normalized to the amount of *β-actin* mRNA. Results are the mean and standard errors from three independent experiments performed in duplicate. * *P* < 0.05 and *** *P* < 0.001 as compared with empty vector.

An inconceivable number of microorganisms live in the intestinal tract of animals and humans [15]. Intestinal epithelial cells participate in the onset and regulation of the intestinal innate immune response to enteric virus infection [16]. During viral infection, IFN-α and IFN-β protect cells against viral invasion by interfering with viral replication. Although STING has been well-characterized as an IFN stimulator since it was cloned in 2008 [11–14], there is no report of its function on intestinal epithelial cells. To examine whether Tibetan porcine STING is capable of stimulating expression of IFN-β in the porcine jejunal epithelial cell line IPEC-J2 cells, the Tibetan porcine *STING* expression plasmid was constructed and transfected into IPEC-J2 cells. Twenty-four hours after the transfection, the mRNA level of Tibetan porcine *STING* in IPEC-J2 cells transfected with the plasmid pcDNA3.1(+)-STING increased 443.56 ± 70.35 times (*n* = 3), compared with cells transfected with the empty vector pcDNA3.1(+) control (data not shown). Thus, the mRNA level of *IFN-β* was tested. As shown in Figure 3, *IFN-β* was significantly upregulated by Tibetan porcine STING overexpression. ISG15 (an IFN-stimulated gene) is strongly upregulated by type I IFN [17]. Here, we showed that ISG15 was also significantly up-regulated by overexpression of Tibetan porcine STING in IPEC-J2 cells (Figure 3). This is the first study investigating the biological role of STING in intestinal epithelial cells, which lays a foundation for the further study of STING in intestinal innate immunity.

## 3. Materials and Methods

### 3.1. Animals and Tissue Sample Collection

Three Tibetan female pigs (body weight of 15.47 ± 0.41 kg) were slaughtered by exsanguination according to protocols approved by the Animal Care Advisory Committee of Sichuan Agricultural University. The heart, liver, lung, spleen, kidney, thymus, small intestine (jejunum), lymph node, skeletal muscle and fat were removed and immediately snap frozen in liquid nitrogen before being stored at −80 °C for RNA isolation.

### 3.2. RNA Isolation and Reverse Transcription

Total RNA was extracted from collected tissue samples using RNAiso Plus reagent (TaKaRa, Dalian, China) according to the manufacturer’s instructions. The concentrations of total RNA were determined spectrophotometrically using a Beckman Coulter DU800 (Beckman Coulter, Fullerton, CA, USA). One microgram of total RNA from each sample was reverse transcribed in a final volume of 20 μL using a PrimeScript^®^ RT reagent Kit with gDNA Eraser (TaKaRa) according to the manufacturer’s protocols. The first-strand cDNA was subsequently used as a template for PCR.

### 3.3. Cloning of STING cDNA

The full coding region of *STING* was obtained by PCR amplification from the total RNA extracted from skeletal muscle of Tibetan pigs. A pair of primers (pMD19-T-STING-F and pMD19-T-STING-R) were designed based on the sequence of Landrace pig *STING* (GenBank ID: FJ455509) and were as follows: 5′-ATGCCCTACTCCAGCCTGCATC-3′ (forward) and 5′-TCAGAAGATATCTGAGCGG AG-3′ (reverse). The PCR was performed in a 50 μL reaction volume containing 2 μL of the first-strand cDNA, 1 μL each of forward and reverse primers from 10 μM stocks, 21 μL DEPC-treated water, and 25 μL of 2 × Taq PCR MasterMix (Tiangen, Beijing, China). The thermal cycling conditions used were: 1 cycle of 94 °C for 3 min, then 35 cycles of 94 °C for 30 s, 63 °C for 30 s, and 72 °C for 1 min 30 s, and followed by 1 cycle of 72 °C for 7 min. The PCR product was purified, cloned into pMD19-T vector (TaKaRa) and sequenced, resulting in pMD19-T-STING.

### 3.4. Plasmid Construction

The DNA fragment containing the entire open reading frame (ORF) of Tibetan porcine *STING* was PCR amplified using the specific primers (pcDNA3.1(+)-STING-F: 5′-TACGAATTCATGCCCTACT CCAGC-3′ and pcDNA3.1(+)-STING-R: 5′-GCTCTAGATCAGAAGATATCTGAGCG-3′) and the plasmid pMD19-T-STING as a template. Primer pcDNA3.1(+)-STING-F introduced an *Eco*RI site, and primer pcDNA3.1(+)-STING-R contained an *Xba*I site (underlined). After digestion with *Eco*RI and *Xba*I, the PCR product was inserted into the vector pcDNA3.1(+) (Invitrogen, Carlsbad, CA, USA). Proper construction was confirmed by sequencing and was designated as pcDNA3.1(+)-STING.

### 3.5. Cell Culture

The IPEC-J2 cell line, originally derived from jejunal epithelia of a neonatal unsuckled piglet [18], was a kind gift from Dr. Junjun Wang (College of Animal Science and Technology, China Agricultural University, Beijing, China). IPEC-J2 cells were maintained in Dulbecco modified Eagle medium (DMEM)/Ham’s F-12 (1:1) medium (Invitrogen) supplemented with 10% fetal bovine serum (FBS) (Invitrogen), and antibiotics (100 U/mL penicillin and 100 μg/L streptomycin) (Invitrogen) at 37 °C, 5% CO_2_ in a humidified atmosphere. Medium was renewed every 2 days.

### 3.6. Transfection

IPEC-J2 cells were seeded in 6-well plates (Corning, NY, USA) at a density of 2.0 × 10^5^ cells/well 24 h prior to transfection. Two micrograms of the plasmid pcDNA3.1(+)-STING or an equal amount of the empty vector pcDNA3.1(+) were transfected using Lipofectamine 2000 (Invitrogen). At 24 h post-transfection, total RNA was extracted and reverse transcribed according to the above-mentioned method. All plasmids were prepared with Endo-free plasmid kit (Omega, Norcross, GA, USA) following the manufacture’s instruction.

### 3.7. Real-Time Quantitative PCR

Real-time quantitative PCR was performed in a CFX96 Real-Time PCR Detection System (Bio-Rad, Hercules, CA, USA). The gene-specific primers used are listed in Table 1. The PCR mixture consisted of 2 μL of the first-strand cDNA sample, 1 μL each of forward and reverse primers from 10 μM stocks, 6 μL DEPC-treated water, and 10 μL of 2 . SsoFast EvaGreen Supermix (Bio-Rad). The PCR cycling conditions used were: 45 cycles of 98 °C for 2 s and 60 °C for 5 s. Each primer pair used yielded a single peak in the melting curve and a single band with the expected size in agarose gel. Identities of the PCR products were confirmed by sequencing. Data were analyzed according to the efficiency-corrected comparative Ct method [19] and were normalized by β-actin expression in each sample.

### 3.8. Statistical Analysis

Data were expressed as mean ± SE. One-way ANOVA and Tukey’s tests (SPSS Inc., Chicago, IL, USA) were performed to assess the statistical significance between treatments. Statistical significance was set at *P* < 0.05.

## 4. Conclusions

Tibetan pig is well known for its strong disease resistance. However, little is known about the molecular basis of its strong resistance to disease. In this study, we first cloned Tibetan porcine *STING*, which encodes a 378-aa protein with 4 transmembrane domains. It is nearly identical to the Landrace pig STING sequence with 12 nucleotide and 5 amino acid differences. Overexpression of Tibetan porcine STING induced *IFN-β* expression. Future studies will be necessary to investigate whether the difference in *STING* sequence is related to the difference in disease resistance ability between Landrace and Tibetan pigs.

## Supplementary Material



## Figures and Tables

**Table 1:** Primers used in real-time quantitative PCR.

Gene	Primer	Sequence	GenBank ID	Product size
*IFN-β*	Forward	5′-AAATCGCTCTCCTGATGTGT-3′	**NM_001003923**	78 bp
	Reverse	5′-TGCTCCTTTGTTGGTATCG-3′		
*ISG15*	Forward	5′-AGCAACGCCTATGAGGTC-3′	**EU584557**	101 bp
	Reverse	5′-AAAGTCAGCCAGAAATGGTC-3′		
*β-actin*	Forward	5′-CCACGAAACTACCTTCAACTCC-3′	**DQ845171**	132 bp
	Reverse	5′-GTGATCTCCTTCTGCATCCTGT-3′		
