# Supplementary material for: Molecular Cloning and Functional Characterization of Tibetan Porcine STING
Source: Int J Mol Sci. 2012 Jan 4;13(1):506–15. doi: 10.3390/ijms13010506 (PMC3269701; doi:10.3390/ijms13010506)
Supplement: Supplementary file 1 [file ijms-13-00506-s001.pdf]

**Figure S1.** Nucleotide and predicted amino acid sequences of Tibetan porcine STING. The numbers on the left refer to positions of the nucleotides or amino acid residues. The stop codon is indicated by *.

```
1       ATGCCCTACTCCAGCCTGCATCCATCCATCCCACAGCCCAGGGGCCTCAGGGCTCAGGAG
1        M  P  Y  S  S  L  H  P  S  I  P  Q  P  R  G  L  R  A  Q  E

61      GCAGCCCTGGTCCTTCTAGGTGCCTGCCTGGTGGCCCTTTGGGGGCTGGGGGAGCTGCCA
21       A  A  L  V  L  L  G  A  C  L  V  A  L  W  G  L  G  E  L  P

121     GAATACACGCTCCGGTGGCTGGTGCTCCACCTGGCCTCCCAGCAGATAGGACTGCTGGTG
41       E  Y  T  L  R  W  L  V  L  H  L  A  S  Q  Q  I  G  L  L  V

181     AAGGGGCTCTGCAGTCTGGCGGAGGAGCTGTGCCACGTCCACTCCAGGTACCAGAGCAGC
61       K  G  L  C  S  L  A  E  E  L  C  H  V  H  S  R  Y  Q  S  S

241     TACTGGAGGGCTGCGTGGGCCTGCCTGGGCTGCCCCATCCGCTGTGGAGCCCTGCTGCTG
81       Y  W  R  A  A  W  A  C  L  G  C  P  I  R  C  G  A  L  L  L

301     CTGTCCTGCTACTTCTACTTCTCCATCCGAGACAAGGCTGGCCTGCCCCTCCCCTGGATG
101      L  S  C  Y  F  Y  F  S  I  R  D  K  A  G  L  P  L  P  W  M

361     CTGGCCCTCCTGGGCCTCTCGCAGGCCCTAAACATCCTCCTGGGCCTCCAGCACCTGGCC
121      L  A  L  L  G  L  S  Q  A  L  N  I  L  L  G  L  Q  H  L  A

421     CCAGCTGAAGTCTCTGCAATCTGTGAAAAAAGGAACTTCAACGTGGCTCATGGACTGGCC
141      P  A  E  V  S  A  I  C  E  K  R  N  F  N  V  A  H  G  L  A

481     TGGTCTTATTACATCGGGTACCTGCGGCTGATCCTCCCAGGGCTCCGGGCCCGGATCCAA
161      W  S  Y  Y  I  G  Y  L  R  L  I  L  P  G  L  R  A  R  I  Q

541     GCTTATAATCAGCGCCACAAGAACGTACTCGGGGGCATAGGGAACCACCGGCTGCACATC
181      A  Y  N  Q  R  H  K  N  V  L  G  G  I  G  N  H  R  L  H  I

601     CTCTTTCCGTTGGACTGTGGGGCGCCCGACGACCTGAGCGTGGCTGACCCCAACATTCGC
201      L  F  P  L  D  C  G  A  P  D  D  L  S  V  A  D  P  N  I  R

661     TTCCTGCACAAGCTGCCCCAGCAAAGTGCCGACCGTGCTGGCATCAAGGGCCGGGTGTAC
221      F  L  H  K  L  P  Q  Q  S  A  D  R  A  G  I  K  G  R  V  Y

721     ACCAACAGCATCTATGAGCTTCTGGAGAACGGGCAGCCGGCAGGCGTCTGTGTCCTGGAG
241      T  N  S  I  Y  E  L  L  E  N  G  Q  P  A  G  V  C  V  L  E

781     TACGCCACTCCCTTGCAGACCTTGTTCGCCATGTCACAGGATGGCCGCGCTGGCTTCAGC
261      Y  A  T  P  L  Q  T  L  F  A  M  S  Q  D  G  R  A  G  F  S

841     CGGGAGGATCGGCTCGAGCAGGCCAAACTCTTCTGCCGGACCCTCGAAGACATCCTGGCA
281      R  E  D  R  L  E  Q  A  K  L  F  C  R  T  L  E  D  I  L  A

901     GATGCCCCTGAGGCTCAGAACAACTGCCGCCTCATCGTCTACCAGGAACCCACAGAGGGA
301      D  A  P  E  A  Q  N  N  C  R  L  I  V  Y  Q  E  P  T  E  G

961     GGCAGCTTCTCCCTGTCGCAGGAGATTCTCCGGCACCTTCGGCAGGAGGAAAGGGAGGTT
321      G  S  F  S  L  S  Q  E  I  L  R  H  L  R  Q  E  E  R  E  V

1021    ACCATGGGCAGTGCGGAGACCTCAGTGGTGCCCACTTCCTCCACGCTGTCCCAAGAGCCT
341      T  M  G  S  A  E  T  S  V  V  P  T  S  S  T  L  S  Q  E  P

1081    GAGCTCCTCATCAGTGGCATGGAACAGCCTCTTCCACTCCGCTCAGATATCTTCTGA
361      E  L  L  I  S  G  M  E  Q  P  L  P  L  R  S  D  I  F  *
```
